# Correlation of cystatin C and creatinine based estimates of renal function in children with hydronephrosis

**DOI:** 10.15171/jrip.2016.06

**Published:** 2016-02-24

**Authors:** Hossein-Emad Momtaz, Arash Dehghan, Mohammad Karimian

**Affiliations:** ^1^Division of pediatric nephrology, Besat Hospital, Hamadan University of Medical Sciences, Hamadan, Iran; ^2^Department of Pathology, Besat Hospital, Hamadan University of Medical Sciences, Hamadan, Iran

**Keywords:** Cystatin C, Creatinine, Hydronephrosis, Renal function

## Abstract

**Introduction:** The use of a simple and accurate glomerular filtration rate (GFR) estimating method aiming minute assessment of renal function can be of great clinical importance.

**Objectives:** This study aimed to determine the association of a GFR estimating by equation that includes only cystatin C (Gentian equation) to equation that include only creatinine (Schwartz equation) among children.

**Patients and Methods:** A total of 31 children aged from 1 day to 5 years with the final diagnosis of unilateral or bilateral hydronephrosis referred to Besat hospital in Hamadan, between March 2010 and February 2011 were consecutively enrolled. Schwartz and Gentian equations were employed to determine GFR based on plasma creatinine and cystatin C levels, respectively.

**Results:** The proportion of GFR based on Schwartz equation was 70.19± 24.86 ml/min/1.73 m^2^, while the level of this parameter based on Gentian method and using cystatin C was 86.97 ± 21.57 ml/min/1.73 m^2^. The Pearson correlation coefficient analysis showed a strong direct association between the two levels of GFR measured by Schwartz equation based on serum creatinine level and Gentian method and using cystatin C (r = 0.594, P < 0.001). The linear association between GFR values measured with the two methods included cystatin C based GFR = 50.8+ 0.515 × Schwartz GFR. The correlation between GFR values measured by using serum creatinine and serum cystatin C measurements remained meaningful even after adjustment for patients’ gender and age (r = 0.724, *P* < 0.001).

**Conclusion:** The equation developed based on cystatin C level is comparable with another equation, based on serum creatinine (Schwartz formula) to estimate GFR in children.

**Implication for health policy/practice/research/medical education:**

In this study we aimed to evaluate the benefit of using serum cystatin C based glomerular filtration rate (GFR) calculation instead of creatinine based formula to early prediction of which hydronephrotic children need surgical intervention. Results of our study showed that in hydronephrotic children, cystatin C based calculation of GFR, is closely related to creatinine based formula but cannot predict early decline of GFR in these patients.

## Introduction

The best reliable method for assessment of renal function is measurement of glomerular filtration rate (GFR) that is defined as the volume of fluid filtered from the renal glomerular capillaries into the Bowman’s capsule per unit time (1). A number of techniques have been introduced for accurate measurement of GFR that among them, applying the clearance of an appropriate chemical marker has priority (2). In this regard, a suitable marker substance which can freely filtered, not be toxic, non-absorbable or secreted in the tubules of the kidney, and also cannot not be altered during passage through the kidneys, will be a suitable substance for assessment (3,4). If the pointed conditions exist, the clearance will be equal to the GFR of the kidneys (5). The gold standard for an appropriate exogenous marker is inulin that is uniquely treated by nephrons and completely filtered at the glomerulus but neither secreted nor reabsorbed by the tubules (6).This property of inulin allows the clearance of inulin to be used clinically as a highly accurate measure of GFR. The measurement of GFR by inulin is still considered the gold-standard. However, it has now been largely replaced by other, simpler measures that are approximations of GFR (7).These measures, which involve clearance of such substrates as EDTA, iohexol, cystatin-C, 125 I-iothalamate, the chromium radioisotope 51Cr (chelated with EDTA), sodium radioiothalamate, and creatinine, have had their utility confirmed in large cohorts of patients with chronic kidney disease (8). Measurement of creatinine clearance is the most widely used approach to estimate GFR. However, according to its changes with aging, and the changes in its renal excretion rate following disease progression, this substance is not appropriate for measuring GFR.According to some major problems in the use of creatinine, other endogenous markers have been recently introduced that among them, cystatin C has been more taken into consideration. It seems that, the changes in this marker are less affected by aging or renal pathological conditions and thus may be more useful than other endogenous markers such as creatinine (9).Most studies have shown that serum levels of cystatin C are more closely correlated with GFR than serum creatinine (10,11).

## Objectives

The purpose of the present study was to determine association of a GFR estimating by equation that includes only cystatin C to equation that include only creatinine.

## Patients and Methods

A total of 31 children aged from 1 day to 5 years with the final diagnosis of unilateral or bilateral hydronephrosis on sonographic assessment who were referred to Besat hospital in Hamadan, were consecutively enrolled to the study. The study population included 21 boys and 10 girls. The study was carried out from March 2010 to February 2011. All subjects met the diagnostic criteria of infantile hydronephrosis, and the following patients were excluded during the selection; 1) patients with known acute kidney disease, acute renal insufficiency, or individuals who underwent dialysis; 2) those with thyroid disorders; 3) children with diabetes mellitus; 4) subjects with malignancies or under treatment with steroids; and finally 5) children with positive urine culture. Baseline information including demographics as well as type and causes of hydronephrosis was collected by interviewing with parents or reviewing hospital recorded files if required. Parents were given a description of the project and then the patients were enrolled in case the parents were satisfied. In order to performing the first step of the study after initial visiting, venous blood samples were obtained from the basilic vein and transferred to hospital laboratory to measure serum level of cystatin C and creatinine. GFR was first calculated using the Schwartz equation (12):

$$eGFR=\frac{k\times height(cm)}{serum\;creatinine(\frac{mg}{dL})}$$

Where, the *k* coefficient is different according to children age group as 0.33 for premature neonates, 0.45 for mature neonates, 0.55 for young children and girls, and 0.70 for young boys.

Once again, Gentian equation was applied to calculate GFR based on serum cystatin C concentration (13):

$$gGFR(\frac{\frac{ml}{min}}{1.73m^{2}})=79.901\times cystatin\;C{\left(\frac{mg}{L}\right)}^{-1.4389}$$

Serum creatinine was measured using the Jaffe method with an alkaline picrate assay. Serum cystatin C was also measured by the rocket electro-immunoassay method using Gentian kit. It should be noted that all tests and the final diagnosis were performed by a specialist and all laboratory specimens were controlled by a single laboratory and by a single machine.

### Ethical issues

1) The research followed the tenets of the Declaration of Helsinki; 2) informed consent was obtained; 3) the research was approved by ethical committee of Hamadan University of Medical Sciences (Approval No. 120136).

### Statistical analysis

Results were reported as mean ± standard deviation (SD) for the quantitative variables and percentages for the categorical variables. The groups were compared using the student’s *t* test or Mann-Whitney U test for the continuous variables and the chi-square test (or Fisher exact test if required) for the categorical variables. The Pearson correlation test was used to determine correlation between the two measuring test. *P* values of 0.05 or less were considered statistically significant. All the statistical analyses were performed using SPSS version 19.0 (SPSS Inc., Chicago, IL, USA) and SAS version 9.1 for Windows (SAS Institute Inc., Cary, NC, USA).

## Results

The mean age of the participants was 6.9 ± 13.9 months ranged 7 days to 60 months and the average of height was 60.6 ± 14.2 cm ranged 48 to 107 cm. The most common reason for hydronephrosis was physiologic in 15 children followed by reflux disease in 10 children and obstructive in other 6 children. In 19 patients, hydronephrosis was appeared as unilateral and in others was occurred bilaterally. The mean level of serum creatinine was 0.50 ± 0.28 mg/dL. The mean serum level of cystatin C was totally 0.98 ± 0.23 mg/L (ranged 0.70 to 1.90 mg/l) that in boys was 1.02 ± 0.12 mg/l and in girls was 0.90 ± 0.10 mg/L with no significant difference (*P* = 0.41). With respect to applying different equations for estimating eGFR, the amount of eGFR based on Schwartz equation was 70.19 ± 24.86 ml/min/1.73 m^2^, while the level of this parameter based on Gentian method and using cystatin C was 86.97 ± 21.57 ml/min/1.73 m^2^. The Pearson correlation coefficient analysis showed a strong direct association between the two levels of eGFR measured by Schwartz equation based on serum creatinine level and Gentian method and using cystatin C (r = 0.594, *P* < 0.001). This association in children younger than 12 months was also significant (r = 0.489, *P* = 0.01). According to this analysis, the following equation was achieved between values ​​measured with the two methods:

Gentian GFR=50.8+0.515×Swartz GFR

The correlation between eGFR values measured by using serum creatinine and serum cystatin C measurements remained meaningful even after adjustment for patients’ gender and age (r = 0.724, *P* < 0.001).

## Discussion

Early detecting impaired renal function based on assessment of GFR is very important because of its development to renal failure, however the overestimation or underestimation of true GFR may lead to insufficient treatment or a scheduling an unnecessary intervention (14). In this regard, the use of a simple and accurate GFR estimating method aiming minute assessment of renal function can be of great clinical importance. Recently, the use of plasma cystatin C based methods has been more paid attention. The plasma level of this agent can increase earlier than some other materials such as serum creatinine and therefore may be more valuable than the latter agent for early detection of renal dysfunction (15,16). The main advantages of plasma cystatin C-based method include freely filtration and catabolizing in the proximal tubule, as well as its independency to patients characteristics of gender, age, or body mass (17). In some studies and meta-analysis, has been reported to be superior to creatinine in GFR estimation, particularly in patients with near-normal kidney function (18-20). In this aim, it seems that the equations developed based on cystatin C level are comparable with or superior to other equations on serum creatinine level that was shown in our study. In a study by Åkerblom and colleagues (21), the overall correlation of Gentian method (based on cystatin C level) and Roche method (based on creatinine level) was 0.86 with high level agreement between them (0.39 mg/l). They also showed high value of Gentian equation for discriminating normal from impaired renal function. In another study by Driver et al (22), the addition of cystatin C can improve mortality risk prediction by stages of kidney function relative to creatinine. According to their observation, kidney disease risk factors were associated with an overestimate of GFR by serum creatinine relative to cystatin C. In another study by Tidman et al (23), it was shown that the estimation of GFR using equations based on serum creatinine or serum cystatin C alone was equally accurate, but the combination of these two could result in a greater accuracy. Also, in a pooled analysis of 3418 individuals with chronic kidney disease, percentages of estimated GFR within 30% of GFR for equations based on serum cystatin C alone, serum cystatin C, serum creatinine, or both levels with age, sex, and race were 81%, 83%, 85%, and 89%, respectively. They finally concluded that the equation using serum cystatin C level alone yields estimates with small biases in age, sex, and race subgroups, which were improved in equations including these variables (24).In a meta-analysis by Zhang et al (25), of 17 studies with 2521 patients with chronic kidney disease, it was demonstrated significant correlations between cystatin C, serum creatinine and GFR. In final, it was shown that GFR estimation on cystatin C was more sensitive, but less specific, than method based on serum creatinine for the estimation of GFR. It seems that GFR estimation is strongly affected by the chosen equation. Along with previous studies, our survey could indicate a strong association between the two estimating equations for determining GFR and assessing renal function in children. Although the value of two equations for determining GFR were equal, because of the independency of cystatin C based method on patients age and gender, the use of this method is preferred to creatinine-based method in children, however, the former method is clearly more expensive and thus creating an equation provided by combination of the two methods not only can lead to more diagnostic accuracy, but also can yield the best cost-benefit ratio for all patients with various economic levels.

## Conclusion

This study shows that in children younger than 5 years old with hydronephrosis, GFR calculated on base of serum cystatin C level has strong statistical correlation with GFR calculated with Schwartz’s formula, however in infants under one year old this correlation seems to be less significant. Although serum cystatin C based formula for calculating GFR in hydronephrotic children is at least as reliable as serum creatinine based Schwartz formula but at present due to higher cost it could not be recommended as a routine or better method of calculating GFR for predicting need of operation in hydronephrotic children.

## Limitations of the study

Small number of patients (31 cases) could be considered a limiting factor in comparison of two methods of calculating GFR in them. We did not analyze cystatin C according to type of hydronephrosis (obstructive versus non-obstructive) which may showed more significant difference between two methods of calculating GFR.

## Acknowledgements

Authors wish to thank and appreciate efforts of Dr. Farzaneh Esniashari for analyzing research data.

## Authors’ contribution

All authors contributed to design of the research. HEM, AD and MK conducted the research. HEM prepared the manuscript. All authors read, revised, and approved the final manuscript.

## Conflict of interests

The authors declare no conflict of interests.

## Ethical considerations

Ethical issues (including plagiarism, misconduct, data fabrication, falsification, double publication or submission, redundancy) have been completely observed by the authors.

## Funding/Support

This study with approval No. 139494 issued by Hamadan University of Medical Sciences. Data collection for this research was supported by vice chancellor of research and technology of Hamadan University of Medical Sciences. The funding sources played no role in the study design, data analysis, and manuscript writing, or in the decision to submit this manuscript for publication.
